# Mitigating infodemics: The relationship between news exposure and trust and belief in COVID-19 fake news and social media spreading

**DOI:** 10.1371/journal.pone.0252830

**Published:** 2021-06-04

**Authors:** Jad Melki, Hani Tamim, Dima Hadid, Maha Makki, Jana El Amine, Eveline Hitti

**Affiliations:** 1 Department of Communication Arts, Institute of Media Research and Training, Lebanese American University, Beirut, Lebanon; 2 Department of Internal Medicine, American University of Beirut Medical Center, Beirut, Lebanon; 3 Department of Emergency Medicine, American University of Beirut Medical Center, Beirut, Lebanon; University of Haifa, ISRAEL

## Abstract

**Introduction:**

Misinformation surrounding COVID-19 poses a global public health problem that adversely affects governments’ abilities to mitigate the disease and causes accidental deaths and self-harm due to false beliefs about the virus, prevention measures, vaccines and cures. We aim to examine the relationship between exposure to and trust in COVID-19 news (from Television, social media, interpersonal communication) and information sources (healthcare experts, government, clerics) and belief in COVID-19 myths and false information, as well as critical verification practices before posting on social media.

**Methods:**

We use a cross-sectional researcher-administered phone survey of adults living in Lebanon between March 27 and April 23, 2020.

**Results:**

The sample included 56.1% men and 43.9% women, 37.9% with a university degree, 63.0% older than 30, and 7% with media literacy training. Those who trust COVID-19 news from social media [95%CI:(1.05–1.52)] and interpersonal communication [95%CI:(1.25–1.82)], and those who trust information from clerics [95%CI:(1.25–1.82)] were more likely to believe in COVID-19 myths and false information. University graduates [95%CI:(0.25–0.51)] and those who trust information from government [95%CI:(0.65–0.89] were less likely to believe in myths and false information. Those who believe in COVID-19 myths and false information [95%CI:(0.25–0.70)] were less likely to engage in critical social media posting practices. Only those who underwent media literacy training [95%CI:(1.24–6.55)] were more likely to engage in critical social media posting practices.

**Conclusion:**

Higher education and trust in information from government contributed to decreasing belief in COVID-19 myths and false information. Trust in news from social media, interpersonal communication and clerics contributed to increasing belief in COVID-19 myths and false information, which in turn contributed to less critical social media posting practices, thereby exacerbated the infodemic. Media literacy training contributed to increasing critical social media posting practices, thereby played a role in mitigating the infodemic.

## Introduction

COVID-19 misinformation and disinformation has spread unabated in the media [1], despite efforts by social media companies to limit its dissemination [2]. UN agencies warned against the plethora of fast spreading false, inaccurate and misleading COVID-19 content [3], which the WHO labelled an infodemic [4]. This included myths and dangerous purported prevention methods and cures that put lives at risk and countered global mitigation efforts [5, 6].

Effective communication remains crucial for mitigating pandemics [7]. Accurate and persistent media coverage increases people’s awareness and comprehension of an outbreak [8–10]. In a media ecosystem characterized by information overload, social media play an equal or greater role than legacy media in informing or misinforming the public about the virus, its diagnosis, prevention methods, and symptoms [11, 12]. Lacking in traditional gatekeeper quality-control mechanisms, social media present a dilemma. Scholars use them to effectively disseminate scientific findings and guidelines [13, 14], while millions peddle multitudes of false information [15, 16], which downplays the disease’s gravity, increases public skepticism about prevention measures and mitigation strategies [17], and promotes dangerous methods and practices [18], particularly in societies with high levels of illiteracy and low levels of media literacy [19, 20].

Several theoretical frameworks have been used to explore trust in media. Selective exposure theory—a concept rooted in Festinger’s [21] theory of cognitive dissonance [22, 23]—posits that audiences seek information consistent with their beliefs to avoid cognitive dissonance, which may lead to psychological stress [22]. A meta-analysis found that audiences were twice as likely to select content that confirms their biases than content that counters it [14, 23]. People tend to perceive attitude-challenging sources as untrustworthy [24] and spend more time on sources they trust [22].

Related to selective exposure theory are the concepts of media trust and source credibility, which explain user susceptibility to messages [25–29]. While individuals’ behavioral responses to imminent threats are influenced by their perception of the severity of the issue [30, 31], this perception is influenced by their trust in these information sources [32]. Audiences more easily comprehend source credibility in branded legacy media (especially Television) than in social media [14], given their hyper diverse, algorithm-driven and user-generated content. Some scholars have associated social media with a rise in polarization and linked them to filter bubbles—algorithmic filters that result from personalized searches that group like-minded people together, and echo chambers—habits that reinforce the beliefs of like-minded individuals [33, 34]. However, other scholars believe that these effects are exaggerated. They argue that diverse information helps users check the information and comprehend other viewpoints, which consequently lessen ideological segregation [35, 36].

Echo chambers may be disrupted through critical media literacy training. Media literacy aims to foster critical thinking among media users and empower individuals with digital skills and social media competencies that help them verify information and disseminate it critically [37]. Media literacy training works effectively as a healthcare and health behavioral intervention, especially when implemented strategically over time in an educational setting [38–42].

Beyond social media and legacy media, research shows that interpersonal communication plays an important role in increasing health awareness and promoting behavioral change in the context of well-understood diseases and campaigns led by healthcare professionals [43, 44]. We know less about the effectiveness of interpersonal communication when knowledge about a disease is still evolving and where in-person contact with healthcare professionals is limited because of disease contagiousness [45], as is the case for COVID-19. In such situations, non-healthcare professionals may fill the knowledge gaps with evolving information that may seem contradictory, which provides fertile grounds for propelling conspiracies, misinterpretations, and disinformation [46].

To our knowledge, studies that have explored media and trust have primarily examined legacy media, and those that included social media preceded the ubiquitous fake news era [11]. In addition, no study has explored the effectiveness of interpersonal communication in the context of a pandemic. We hypothesize that trust in and exposure to social media and interpersonal communication news about COVID-19 will be (a) positively associated with belief in myths and false information about COVID-19 and (b) negatively associated with critical verification practices for social media sharing. We also postulate that media literacy as well as trust in information from (a) healthcare experts and (b) government will be negatively associated with belief in COVID-19 myths and false information and positively associated with critical verification practices for social media sharing, while the opposite will be true for trust in information from (c) clerics. Clerics and religious institutions have undeniable influence on political and social matters in Lebanon—including healthcare; so, we included clerics in the study as a major source of information along with government and healthcare experts. Lastly, we hypothesize that belief in COVID-19 myths and false information will be negatively associated with critical verification practices for social media sharing.

This study, therefore, examines the role of legacy media and social media, as well as interpersonal communication, in mitigating infodemics, and uses robust theoretical constructs from communication, including selective exposure, media trust, and media literacy. It examines the relationship between media exposure and trust and the belief in COVID-19 myths and false information, and the critical verification practices people follow before posting on social media.

## Methods

### Study design

A cross-sectional phone survey was administered to adults residing in Lebanon between March 27 and April 23, 2020. Phone surveys were deemed most feasible at the time of the study given the need for social distancing. The questionnaire encompassed 56 close-ended questions and required approximately 15–20 minutes. IRB approved the study under protocol number LAU.SAS.JM1.20/Mar/2020.

### Study setting

The study was conducted in Lebanon, a low-middle income Arab country, with an estimated 6 million residents, including 1.5 million displaced individuals and 500,000 migrant workers. The first case of COVID 19 was identified on February 21^st^, a few weeks after a newly appointed government had taken power, following political and financial turmoil that brought down the previous cabinet and pushed the country into an economic crisis. Lebanon’s initial COVID-19 response during the study period followed a stringent containment effort to allow for capacity building and preparedness in the health sector. This was led by the National COVID-19 Committee (NCC), a committee comprised of academics, healthcare professionals, governmental representatives, and representatives of the major non-governmental organizations including UN agencies, the WHO and the Red Cross. The measures included early closure of schools, daycare centers, and universities, followed by complete shutdown of non-essential services, in addition to border closure when total cases were still below 100. In addition, the NCC implemented an aggressive communication campaign, flooding media outlets with information from healthcare professionals and governmental prevention directives. This led to high knowledge levels on prevention and self-reported adherence to governmental recommendations, including hand hygiene, avoiding crowds, and staying at home [47].

### Participants and sample size calculation

We calculated a sample size of 1,536 based on a population of 6M (95%CI, ±2.5%) and developed a proportional random sample based on all possible mobile number ranges in Lebanon. We obtained all possible number ranges from the Ministry of Telecommunication, developed a sampling frame based on these number ranges, and selected proportional random samples from each range. Invalid phone numbers and non-responders (after five attempts) were replaced up to two times. Respondents older than 18 were verbally consented prior to administering the survey. We excluded questionnaires less than 85% complete and did not replace refused responses. The final tally of 792 responses generated a 51.6% response rate.

### Measurements

#### Media trust

To assess media trust for television, social media, and interpersonal communication, participants were asked how often they trust COVID-19 news from these sources and responded to a 4-point scale (never, rarely, sometimes, and often). We used the same measures for assessing trust in three main information sources: healthcare experts, government, and clerics.

#### Media exposure

To assess media uses of television, social media, and interpersonal communication, participants were asked “How often did you follow Coronavirus news through the following?” The responses were also measured on a 4-point Likert scale of “never,” “rarely,” “sometimes,” and “often.”

#### Myths and false information

To assess belief in COVID-19-related myths, conspiracies, and false information, participants were asked about their prevention behavior: “How often do you do the following as a prevention measure for Coronavirus?” Responses were measured on the same 4-point scale, as media trust. The list of prevention activities included true options (avoid crowds, wash hands with water and soap, stay at home, avoid shaking hands, avoid touching my face, cover my cough) and false options (eat garlic or bananas, wash inside my nose with warm salty water). Participants were also asked whether they agreed or disagreed with the following statements, using a 4-point Likert scale (strongly disagree, somewhat disagree, somewhat agree, strongly agree): “Coronavirus is a man-made biological weapon,” “Coronavirus is created by pharmaceuticals for profit purposes,” and “Coronavirus is a punishment from God.” Finally, participants answered if the following groups are “at high risk of dying from Coronavirus” with yes or no. The list included a mix of widely circulating false and prejudiced information (children younger than 8; Chinese and Iranians; those with blood type O-positive) and correct answers (Older people above 65; people with diabetes, heart or lung diseases; immunodeficient individuals). We developed a combined scale out of all the false answers. False answers were based on widespread false beliefs and practices in the country that were extracted from the media at the time of the study.

#### Critical social media posting practices

Social media posting was first filtered through the question “How often did you post Coronavirus news on social media?” and the responses: never, rarely, sometimes, often). Those who selected at least “rarely” were asked “How often did you do the following before posting or sharing news about Coronavirus on social media?” Using the same 4-point response scale, participants responded to the following options: “I checked the original source of the news before posting,” “I compared the information to an expert or credible source before posting,” and “I published most news I received about Coronavirus.” We developed a combined scaled for the first two statements and the reverse of the third statement.

#### Media literacy and demographics

In addition to demographics, we asked participants if they received any media literacy training in their lives.

The Likert scale scores for all of the above variables were transformed into 0–100, where a score of 0 indicates an incorrect answer and a score of 100 indicates a correct answer.

### Statistical analysis

The participants were divided into groups according to their median level of belief in myths and false information as well as engagement in critical verification practices for social media sharing. Those lower than the median were considered to have less belief in myths and false information and less engagement in critical verification practices for social media sharing, whereas those above the median were considered to have greater belief in myths and false information and good engagement in critical verification practices for social media sharing.

The statistical Package for Social Sciences (SPSS), version 24, was used to perform data management and analyses. We presented the distributions of the continuous and categorical variables as means/standard deviations and frequencies/percentages, respectively. We carried out Chi-square tests to examine the association between *myths and false Information* levels or *critical social media practices* and other categorical variables and used t-tests for continuous variables. We deployed multivariate regression analyses to adjust for potentially confounding variables. The forward stepwise logistic regression analysis assessed the association between the two outcomes (COVID-19 myths and false information, and critical verification practices for social media sharing) and the different predictors where the p-value for entry was 0.05 and that for removal was 0.10 P-value ≤ 0.05 indicated statistical significance.

## Results

Table 1 presents demographic characteristics: 56.1% men, 43.9% women, 37.9% completed a university degree, 63.0% older than 30, and 7% underwent media literacy training.

**Table 1:** Sample demographic characteristics.

Media Exposure (TV/Social media)		
		Total
		N = 792
**Gender**	Men	443 (56.1)
	Women	346 (43.9)
**Age**	≤30	288 (37.0)
	>30	491 (63.0)
**Education**	<University	477 (62.1)
	University	291 (37.9)
**Media Literacy**	No	736 (93.0)
	Yes	55 (7.0)

Table 2 presents the association between participants’ characteristics, exposure to media, and trust in media and information sources with the first outcome: Belief in COVID-19 myths and false information. Participants scoring lower than the median are less likely to believe in myths and false information, whereas those scoring above the median are more likely to do so. Those who believe in COVID-19 myths and false information are slightly more likely to be women (47.6% vs 40.7%, p = 0.04), and much more likely to have less than a university education (76.8% vs 49.4%, p<0.0001). Trust in COVID-19 news from Television (3.20±0.86 vs 3.06±0.89, p = 0.03), social media (2.59±0.94 vs 2.24±0.88, p<0.0001), interpersonal communication (2.51±0.96 vs 2.02±0.88, p<0.0001), and clerics (2.78±1.21 vs 2.23±1.19, p<0.0001) positively associated with belief in COVID-19 myths and false information. Exposure to Television (3.50±0.80 vs 3.35±0.97, p = 0.02) and interpersonal communication (2.86±1.11vs 2.59±1.20, p = 0.001) revealed similar associations.

**Table 2:** Association of trust in media and information sources with myths and false information.

	Belief in COVID-19 Myths and False Information score		
	**score≤41.67**	**score>41.67**	**p-value**
	**N = 430**	**N = 362**	
**Age**			
≤30	146 (34.8)	142 (39.6)	0.17
>30	274 (65.2)	217 (60.4)	
**Gender**			
Men	254 (59.3)	189 (52.4)	0.04
Women	174 (40.7)	172 (47.6)	
**Education**			
Less than university graduate	203 (49.4)	274 (76.8)	<0.0001
University graduate and above	208 (50.6)	83 (23.2)	
**Media literacy**	33 (7.7)	22 (6.1)	0.37
**Information & Media Trust**			
Television	3.06 ± 0.89	3.20 ± 0.86	0.03
Social media	2.24 ± 0.88	2.59 ± 0.94	<0.0001
Interpersonal communication	2.02 ± 0.88	2.51 ± 0.96	<0.0001
Healthcare experts	3.59 ± 0.63	3.58 ± 0.66	0.92
Government	3.04 ± 1.11	2.95 ± 1.15	0.28
Clerics	2.23 ± 1.19	2.78 ± 1.21	<0.0001
**Media Exposure**			
Television	3.35 ± 0.97	3.50 ± 0.80	0.02
Social media	3.20 ± 1.03	3.27 ± 1.05	0.31
Interpersonal communication	2.59 ± 1.20	2.86 ± 1.11	0.001

Table 3 presents the association between participants’ characteristics, exposure to media, trust in media and information sources, and belief in myths and false information with the second outcome: Engagement in critical verification practices for social media sharing. Participants scoring lower than the median are less likely to engage in critical verification practices before sharing information on social media, whereas those scoring above the median are more likely to do so. Those with a university education (60.5% vs 45.8, p = 0.02) and those who have undergone media literacy training (19.5% vs 6.5%, p = 0.001) are more likely to engage in critical verification practices for social media sharing. Trusting social media (2.66±0.85 vs 2.35±0.86, p = 0.004), interpersonal communication (2.43±0.91 vs 2.18±0.89, p = 0.03), medical experts (3.64±0.61 vs 3.49±0.66, p = 0.05) and clerics (2.58±1.21 vs 2.29±1.11, p = 0.04) negatively associated with critical verification practices for social media sharing. Consistently, overall belief in COVID-19 myths and false information negatively associated with critical verification practices for social media sharing (45.79±18.16 vs 34.53±17.37, p<0.0001).

**Table 3:** Association of trust in media/information sources and belief myths/false information with critical verification practices for social media sharing.

	Critical verification practices for social media sharing score		
	**score≤3**	**score>3**	**p-value**
	**N = 155**	**N = 119**	
**Age**			
≤30	72 (47.1)	50 (42.4)	0.44
>30	81 (52.9)	68 (57.6)	
**Gender**			
Men	79 (51.0)	62 (52.1)	0.85
Women	76 (49.0)	57 (47.9)	
**Education**			
Less than university graduate	84 (54.2)	47 (39.5)	0.02
University graduate and above	71 (45.8)	72 (60.5)	
**Media literacy**	10 (6.5)	23 (19.5)	0.001
**Information & Media Trust**			
Television	3.18 ± 0.86	3.12 ± 0.84	0.54
Social media	2.66 ± 0.85	2.35 ± 0.86	0.004
Interpersonal communication	2.43 ± 0.91	2.18 ± 0.89	0.03
Healthcare experts	3.64 ± 0.61	3.49 ± 0.66	0.05
Government	2.92 ± 1.09	3.07 ± 1.10	0.28
Clerics	2.58 ± 1.21	2.29 ± 1.11	0.04
**Media Exposure**			
Television	3.41 ± 0.87	3.32 ± 0.93	0.39
Social media	3.50 ± 0.81	3.46 ± 0.82	0.68
Interpersonal communication	2.92 ± 1.09	2.76 ± 1.12	0.26
**Outcome**			
Belief in myths and false information	45.79 ± 18.16	34.53 ± 17.37	<0.0001

Table 4 presents the predictors of both outcomes: belief in COVID-19 myths and false information and engagement in critical verification practices for social media sharing. Those who trust COVID-19 news from social media [OR = 1.25, 95%CI:(1.05–1.52), p = 0.02] and interpersonal communication sources [OR = 1.51, 95%CI:(1.25–1.82), <0.0001], and those who trust information from clerics [OR = 1.28, 95%CI:(1.11–1.49), p = 0.001] were more likely to believe in COVID-19 myths and false information. Women [OR = 1.41, 95%CI:(1.01–1.97), p = 0.04] were also slightly more likely to do so. University graduates [OR = 0.36, 95%CI:(0.25–0.51), p<0.0001] and those who trust information from the government [OR = 0.76, 95%CI:(0.65–0.89, p<0.0001] were less likely to believe in COVID-19 myths and false information. Those who believe in COVID-19 myths and false information are less likely to engage in critical verification practices for social media sharing [OR = 0.41, 95%CI:(0.25–0.70), p = 0.001]. Only those who underwent media literacy training were more likely to engage in critical verification practices for social media sharing [OR = 2.85, 95%CI:(1.24–6.55), p = 0.01].

**Table 4:** Stepwise logistic regression of the predictors of the first (Belief in COVID-19 myths and false information) and second (critical verification practices for social media sharing) outcomes.

	OR (95%CI)	P-value
**Belief in COVID-19 myths and false information score (reference: score≤41.67)**		
Gender–women	1.41 (1.01–1.97)	0.04
Education–university graduate	0.36 (0.25–0.51)	<0.0001
Social media (Trust)	1.25 (1.05–1.52)	0.02
Interpersonal communication (Trust)	1.51 (1.25–1.82)	<0.0001
Government (Trust)	0.76 (0.65–0.89)	<0.0001
Clerics (Trust)	1.28 (1.11–1.49)	0.001
**Critical verification practices for social media sharing score (reference: score≤3)**		
Media Literacy	2.85 (1.24–6.55)	0.01
**Belief in COVID-19 myths and false information score (score>41.67)**	0.41 (0.25–0.70)	0.001

Variables included in the model: gender (reference: men), age (reference: <30), education (reference: <university), media literacy, Television, social media, interpersonal communication (trust and exposure), healthcare experts, government, clerics, myths and false information (reference: score≤ 41.67 (median)) adjusted for critical practices outcome.

## Discussion

This study shows that those who trust news from social media, interpersonal communication, and clerics are more likely to believe in COVID-19 myths and false information, while those with higher education and those who trust government information sources are less likely to believe in COVID-19 myths and false information. Women were also slightly more likely than men to believe in such myths. In addition, belief in COVID-19 myths and false information contributes to less engagement in critical verification practices for social media sharing. Lastly, our results show that only media literacy training predicts higher likelihood of engaging in critical verification practices for social media sharing, which emphasizes the importance of media literacy training in mitigating the infodemic.

This is a first of its kind study to be conducted during a pandemic and to examine the relationship between media exposure and trust and the belief in COVID-19 myths and false information, as well as critical verification practices before posting on social media. Nearly all research on media uses and trust during pandemics studied the 2009 H1N1 Flu [30, 45] and none has studied Arab countries. Prior studies primarily examined legacy media and disregarded interpersonal communication [45], and those that included social media preceded the ubiquitous fake news era [11].

Our study found that trust in COVID-19 news from social media and interpersonal communication (but not Television) predicted belief in COVID-19 myths and false information but did not explain critical verification practices for social media sharing. First, trusting news from social media may be inversely related to critical thinking, particularly in terms of verifying and filtering information from this rich media source. Given that trusting Television—whose information is filtered and vetted by traditional gatekeepers and which in Lebanon was consistent with government directives at the time of the study—did not predict believing in COVID-19 myths and false information, media trust and selective exposure theory here explain that audiences who already believed in these myths confirmed their beliefs through biased social media sources [22, 27], while those who were uncertain of their attitude (and may have not believed in these myths) were willing to gather information that may contradict their (uncertain) beliefs in order to develop their attitude [48]. This is consistent with previous research that found that frequent exposure to false news increases the probability of individuals’ acceptance of [14] and susceptibility to such news [14, 49]. Second, for interpersonal communication, our findings raise a cautionary note about relying on interpersonal discussions and campaigns that exclude healthcare professionals, particularly for emerging diseases where knowledge about them is evolving and changing [45], especially for individuals with low education [43, 50] and for diseases prone to stigmatization [51], as is the case for COVID-19.

Our findings also show that trust in COVID-19 information from government contributed to reducing belief in myths and false information, which is surprising since we assumed that the severe economic and political crises in the country had lowered the level of trust in government [52]. However, this may be explained through the strong health communication strategy followed at the time, which included a unified public health message emanating from a committee that included public health and government officials, healthcare professionals and communication experts with reputable academic backgrounds. This finding confirms previous studies that found a positive relationship between trust in government and better health, as well as less trust-related barriers to healthcare services [53]. The point is worth future exploration, given how major developed countries, including the US and UK, struggled with mitigating the pandemic—and mixed messaging from governmental agencies may have contributed to the plummeting trust in information from these governments [47, 54]. In addition, given the powerful influence of clerics and religious institutions in Lebanon and our finding that those who trust clerics are more likely to believe in COVID-19 myths and false information, it is pertinent to further explore the influence of different religious institutions. Emerging research shows that certain conservative religious groups in the US are more likely to believe in false information surrounding COVID-19 vaccination [55]. We would assume that investing in the training of clerics on scientific literacy surrounding COVID-19 and incorporating their service into national communication strategies would yield positive results in fighting the pandemic. The strategy of engaging clerics has been used recently to varying levels of success in the arduous mission of countering religious extremism. Engaging clerics in national healthcare emergency plans should be less tedious and more likely to yield positive results.

When it comes to gender, our finding that women were slightly more likely to believe in COVID-19 myths and false information is consistent with emerging research about COVID-19 and fake news [56]. This may be explained by the “news consumption gender gap” phenomenon, where women are less likely than men to consume news [57] and more likely to avoid it [58]. Such gender gap is attributed to a perception that news is created mainly for men and along with the unequal domestic burdens women shoulder in most societies, which preclude them from dedicating significant time and attention to news consumption. These “domestic tethers” have also contributed to the gendered journalism environment in Lebanon, where men dominate decision-making positions in the news industry, thereby exacerbating the “news for men” perception [59].

Moreover, belief in COVID-19 myths and false information was negatively associated with critical verification practices for social media sharing, which we believe contributed to a vicious cycle of spreading further false information on social media and to more belief in such information for those who trust social media. This confirms the global call to mitigate the infodemic behind the pandemic [3–6].

While media literacy training did not relate to belief in COVID-19 myths and false information, it was the only predictor of critical verification practices for social media sharing. We believe the former was not supported because it is content-related (health literacy), and media literacy curricula do not always cover health issues, while the latter is skills-related (information verification), and media literacy information-verification skills are transferable to various contexts [37]. Furthermore, media literacy does not preach distrusting media but critical media consumption [60], although sometimes the outcomes lead to cynicism and distrust [61]. This finding requires further examination using more sophisticated media literacy measures to pinpoint specific media literacy competencies that contribute to critical social media posting practices. Nevertheless, given that education level was negatively related to belief in COVID-19 myths and false information, we believe that integrating media literacy courses in school and university curricula with strong health literacy modules will significantly help counter infodemics.

### Limitations

Use of a phone surveys may have missed a select demographic that does not own phones. However, phone ownership in Lebanon is high with 88% of Lebanese owning a mobile device [62]. Another limitation is that survey methodology captured self-reported behavior not actual behavior. We also note the following limitations: 27.2% (418) refused to participate, 18.2% (279) of phone numbers were not valid or did not answer, and 3% (47) did not sufficiently complete the questionnaire. Finally, although participants’ geographical distribution was largely proportional to the actual population distribution, three governorates were slightly underrepresented, whereas the capital Beirut was somewhat overrepresented. This however mirrors the fact that most Lebanese residing in these governorates work and live in Beirut during the week.

## Conclusion

Higher education and trust in information from government contributed to decreasing belief in COVID-19 myths and false information, while trust in news from social media, interpersonal communication and clerics did the opposite. Belief in COVID-19 myths and false information contributed to less critical verification practices before posting on social media, thereby contributed to a cycle of spreading myths and false information on social media. In contrast, media literacy training contributed to breaking that cycle by increasing critical verification practices before sharing on social media, thereby contributed to mitigating the infodemic.

## Supporting information

S1 File This is the de-identified dataset. (SAV)
